# Physical Activity and Long-Term Risk of Breast Cancer, Associations with Time in Life and Body Composition in the Prospective Malmö Diet and Cancer Study

**DOI:** 10.3390/cancers14081960

**Published:** 2022-04-13

**Authors:** Öykü Boraka, Marie Klintman, Ann H. Rosendahl

**Affiliations:** Department of Clinical Science Lund, Oncology, Lund University and Skåne University Hospital, 221 85 Lund, Sweden; oyku.boraka@med.lu.se (Ö.B.); marie.klintman@med.lu.se (M.K.)

**Keywords:** breast cancer, physical activity, menopausal status, body composition

## Abstract

**Simple Summary:**

Regular physical activity has long been recognized as an important preventive measure against many chronic diseases including breast cancer. Whether the benefits differ by time in life or body composition are yet to be answered. With this research, we aimed to expand the knowledge regarding the association between physical activity and breast cancer risk. We confirmed an overall 23% lower long-term breast cancer risk among women engaging in high (corresponding to >1 h daily walking per week) vs. low physical activity and showed that physical activity exerts its greatest benefits to women during or after menopause, or among women with body compositions (waist circumference, body fat, or BMI) in the upper-normal-to-overweight range.

**Abstract:**

Being physically active as part of everyday life reduces breast cancer risk. Less is known whether the benefits of an active lifestyle differ depending on the timing of physical activity in life or anthropometric characteristics. The aim of this study was to bring further insights to the association of physical activity in relation to menopausal status and body composition with breast cancer risk by making use of a prospective Swedish cohort (Malmö Diet and Cancer Study) with long-term follow-up. Physical activity information of 15,983 participants for the past 12 months prior to study entry was assessed according to metabolic equivalent task (MET)-hours/week to integrate duration and intensity of reported activities. During 23.2 years median follow-up, 1302 invasive breast cancers occurred. Women reporting a high physical activity at study baseline, corresponding to >1 h daily walking/week (≥28.5 MET-h/week), had a 23% lower long-term breast cancer risk (HR_adj_ = 0.77, 95% CI 0.66–0.90) than those reporting low physical activity, being most pronounced among perimenopausal and postmenopausal women, and women with waist circumference, body fat percentage, or BMI in the upper-normal and overweight range. For premenopausal women or women having obesity or the largest body composition, high physical activity alone did not modify the breast cancer risk, suggesting additional preventive measures indicated in these groups to reduce the long-term risk of breast cancer.

## 1. Introduction

Female breast cancer has become the most frequently diagnosed cancer in the world with 2.26 million new cases in 2020 and remains the leading cause of cancer death among women [1]. The almost-steady increase in breast cancer incidence in the transitioned countries has in part been attributed to the lifestyle changes in parallel to the socio-economic developments that have occurred in the past century, especially since the 1980s [1], such as less and/or late reproduction, alcohol consumption, a diet rich in energy and fatty acids, and low levels of physical activity [2]. The latter two lifestyle changes have also contributed to the emergence of an overweight and obesity epidemic [3] in which globally, 39% and 13% of adults are estimated to be overweight and obese, respectively [4]. The definitions of overweight and obesity are determined by the World Health Organization (WHO) with the use of body mass index (BMI) as a measure of weight-to-height, with individuals who have a BMI of 25.0–29.9 kg/m^2^ and ≥30.0 kg/m^2^ referred to as overweight and obese, respectively [4]. Having a high BMI, through evidence from observational studies, has been established as a risk factor for developing a wide variety of health conditions, such as cardiovascular diseases [5], type 2 diabetes [6], clinical depression [7], and certain cancer types including postmenopausal breast cancer [8]. The association between high BMI and an increased risk of postmenopausal breast cancer has been postulated in part via the elevated levels of estrogen due to its biosynthesis in the adipose tissue [9]. In premenopausal women, having a high BMI has been reported to be neutrally or inversely associated with breast cancer risk, although subtype-specific differences have been reported [10,11,12].

Leisure-time physical activity has, in addition to achieving and maintaining a healthy weight, been associated with a lower risk of at least 13 different types of cancer, including breast cancer, and may relate to its modulation of certain hormones (e.g., estrogen, insulin, insulin-like growth factors, and various adipokines) and the immune system [13,14,15]. The inverse association between physical activity and breast cancer risk has been reported for both pre- and postmenopausal breast cancer [16,17,18], although, the evidence is less for premenopausal cancer than its postmenopausal counterpart [17,19]. Mixed results on the modifying effects of BMI exist with regard to physical activity and breast cancer risk, and few studies have examined associations with adiposity measures beyond BMI [13,20]. Between 2013–2016, the overall proportion of breast cancer cases in the USA attributable to physical inactivity was estimated to be 6.3% [21]. Regular physical activity has been recommended as a preventive measure against breast cancer by the WHO which, when taken together with all other recommended measures (e.g., weight control, avoidance of alcohol and tobacco, and prolonged hormone use), bears the estimated potential to decrease breast cancer risk by up to 30% [22].

Despite the associations established so far, more links between physical activity and breast cancer risk reduction are yet to be elucidated to fill the knowledge gap whether the benefits vary with regard to time in life and body composition. These novel understandings are necessary in order to intervene and influence in a beneficial direction for the better health of women. The aim of this study was to investigate the associations between physical activity and breast cancer risk in relation to menopausal status and body composition assessed by waist circumference, body fat percentage, and BMI, by making use of a prospective Swedish cohort with long-term follow-up.

## 2. Materials and Methods

### 2.1. Study Population

The participants in the study population were recruited in Malmö, Sweden, from 1991–1996 for the observational Malmö Diet and Cancer Study (MDCS) that aimed to elucidate potential associations between dietary habits and cancer development in a prospective manner. Residents of Malmö who were born between 1923–1950 were invited to the study, and a total of 28,098 participants, of whom 17,035 were women, were enrolled and provided with a written informed consent. At study entry, baseline data regarding socioeconomic status, lifestyle (including physical activity) and reproductive factors of the participants were collected through a self-reported questionnaire, and anthropometric measurements were taken by trained research nurses. Ethical approvals for the MDCS (LU 51-90) and the present study (Dnr 652/2005, 2014/830) were obtained from the regional ethics committee in Lund.

### 2.2. Eligibility and Exclusion Criteria

**Figure 1:**
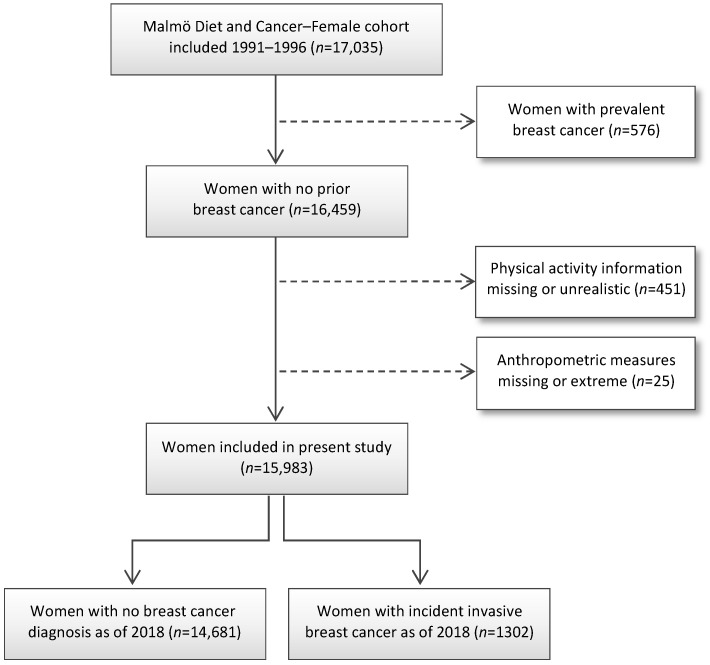
CONSORT flow diagram of the study population.

Women were excluded from the present study if having prevalent breast cancer (*n* = 576), missing or unrealistic physical activity reports (*n* = 451, including two individuals with >16 h activity/day), and missing or extreme anthropometric measures (*n* = 25, including one woman with a waist circumference of 50 cm and a BMI of 34.6 kg/m^2^, and one woman with a waist circumference of 841 cm). The remaining 15,983 women were included in the present study (Figure 1).

### 2.3. Physical Activity Assessment

Participating women provided detailed information through self-reported questionnaires regarding their weekly physical activity in leisure time and movement to and from work over the four seasons during the preceding year prior to study entry. The physical activity questionnaire applied in the MDCS was adapted from a modified Minnesota Leisure Time Physical Activity questionnaire [23] and has later been validated against an accelerometer monitoring at a reexamination of a random subgroup of 369 male and female MDCS participants [24]. In total, 17 types of activities were recorded, including badminton, ballroom dancing, cycling, digging, folk dancing, gardening, golf, grass cutting, jogging, keep-fit exercise, orienteering, soccer, swimming, table tennis, tennis, walking, and walking stairs. The level of physical activity was computed according to international standards for metabolic equivalent task (MET)-hours per week to incorporate the duration and intensity of reported activities. For each activity, the average hours per week that were spent across all seasons were multiplied with their corresponding metabolic equivalents value according to the 2011 Adult Compendium of Physical Activities [25]. The weekly MET-hours for all 17 types of activities were then summarized for each participant. Finally, based on the median of total MET-hours per week (MET-h/week) in the study population, physical activity levels were dichotomized as low (<28.5 MET-h/week) or high (≥28.5 MET-h/week), equivalent to less or more than one hour of daily walking per week, respectively.

### 2.4. Definition of Subgroups by Menopausal Status and Body Composition

Women were defined according to their menopausal status at baseline and categorized as premenopausal, perimenopausal, and postmenopausal. Women were considered postmenopausal if having undergone bilateral oophorectomy or if menstruation had ceased >2 years prior to baseline; perimenopausal if menstruation had ceased <2 years prior to baseline. If the above information was unavailable (<2% of the women), the women were considered postmenopausal if 55 years or older at baseline; perimenopausal if 42–54 years at baseline. The remaining women were classified as premenopausal. Participants were further described by four categories of waist circumference: lowest (<73.0 cm), lower-middle (73.0–76.9 cm), upper-middle (77.0–83.9 cm), and highest (≥84.0 cm), corresponding to European clothing sizes small, medium, large, and extra-large, respectively. BMI was categorized into lower-normal (<23.0 kg/m^2^), upper-normal (23.0–24.9 kg/m^2^), overweight (25.0–29.9 kg/m^2^), and obese (≥30.0 kg/m^2^). Body composition measures of total body fat mass were recorded using Bioelectrical Impedance Analyzer BIA 103 (RLJ Systems, Clinton Township, MI, USA), with body fat percentage calculated as 100 * (fat weight/weight). Body fat percentage was categorized into lowest (<28.0%), lower-middle (28.0–31.9%), upper-middle (32.0–34.9%), and highest (≥35.0%), approximating body fat classifications for women aged 50–69 as good, fair, poor, and high, respectively.

### 2.5. Endpoint

Information and date of breast cancer diagnosis was obtained from the Swedish Cancer Register. Emigration was identified through the Swedish Population Register. Date of death was obtained from the Swedish Cause of Death Register. The primary endpoint and indicator of event for the present study was incident invasive breast cancer. Participants were followed from the date of study entry to the date of breast cancer diagnosis, and were censored in the event of emigration, death, or end of follow-up until 31 December 2018.

### 2.6. Statistical Analysis

The incidence of invasive breast cancer was calculated per 100,000 person-years (BCs/100,000 py) as an estimate of absolute risk according to the level of physical activity for all women and stratified by menopausal status or anthropometric measures. The relative risk of invasive breast cancer comparing high vs. low levels of leisure time physical activity was calculated and expressed as a difference in percentage. The Kaplan–Meier curve and LogRank test were used to compare differences between groups of women with high or low physical activity, and to visually evaluate the proportional hazards assumption. Due to signs of assumption violation in the early period, LogRank test and subsequent analyses were performed both for the complete follow-up period and for the early (0–11 years) and late (12+ years) periods, separately. Age-adjusted and multivariable-adjusted Cox regression analyses providing hazard ratios (HR) with 95% confidence interval (95% CI) for breast cancer risk according to high vs. low levels of physical activity were performed with duration of follow-up as the underlying timescale.

#### Covariate Adjustments

Subgroup analyses for menopausal status, waist circumference, BMI, and body fat were also conducted. The multivariable model was adjusted for age at baseline (continuous), age at menarche (≤12 years, 13–14 years, >15 years), parity (0, 1, 2, 3, 4, or more), age at first childbirth (nulliparous, ≤20 years, 21–25 years, 26–30 years, >30 years), oral contraceptive use (never, ever), current hormone replacement therapy (no, yes), socioeconomic index (manual worker, nonmanual worker, employer/self-employed), and alcohol consumption (nothing last year, something last year, something last month). Models for all women or stratified by body composition (waist circumference, BMI, or body fat percentage) were additionally adjusted for age at menopause (pre/perimenopausal, hysterectomy/bilateral oophorectomy, postmenopausal ≤44, 45–54, ≥55 years). All statistical analyses were conducted on SPSS (IBM Version 27 for Mac).

## 3. Results

### 3.1. Baseline Characteristics Based on Physical Activity Levels

The baseline characteristics were similar overall between women who were included in the study and engaged in low levels (<28.5 MET-h/week, *n* = 7990) or high levels (≥28.5 MET-h/week, *n* = 7993) of physical activity (Table 1). The median age at baseline was 56.6 and 56.3 years for low and high physical activity groups, respectively. Women who were not included in the study were older at baseline, younger at first breast cancer diagnosis, more frequent manual workers, and less likely to have had children compared to women who were included. Among the included study participants, anthropometric measures and reproductive factors were similar across low and high physical activity groups, whereas the low physical activity group had a higher frequency of manual workers and women who did not consume alcohol in the past year than the high physical activity counterpart.

### 3.2. Physical Activity Levels in Relation to Breast Cancer Status

Physical activity levels were lower among women who developed breast cancer compared to women who did not, both in min/week (353 (interquartile range; IQR 222–555) vs. 380 (IQR 228–580), respectively) and MET-h/week (26.7 (IQR 17.0–42.0) vs. 28.6 (IQR 17.3–43.9), respectively) (Table 2). The differences in physical activity levels between women who developed breast cancer or not were most pronounced in perimenopausal and postmenopausal women, among women who had lower-middle and upper-middle waist circumference, in women with upper-normal and overweight BMI, and lower-middle and upper-middle body fat percentages.

### 3.3. Breast Cancer Incidence Based on Physical Activity Levels

The median follow-up time was 23.2 years (IQR 19.1–25.2) and median time to breast cancer diagnosis was 12.2 years (IQR 6.9–17.6). During follow-up, 1302 women among 15,983 participants had developed breast cancer. Of the women diagnosed with breast cancer, 699 were in the low physical activity group and 603 in the high physical activity group (Table 3). Overall, the breast cancer incidence per 100,000 person-years was 418 and 356 for low and high physical activity, respectively, corresponding to the relative risk of developing breast cancer to be reduced by 14.9% with high physical activity compared to low physical activity. Considering menopausal status, the most pronounced reductions in relative risk were observed among peri- (−34.4%) and postmenopausal (−16.7%) women. In relation to body composition, the largest difference in absolute and relative risk of breast cancer when comparing high vs. low physical activity were found among women with lower-middle (−33.1%) and upper-middle (−30.0%) waist circumferences, upper-normal (−20.6%) and overweight (−19.7%) BMI levels, and lower-middle (−20.2%) and upper-middle (−23.1%) body fat percentages (Table 3). Premenopausal women and women who had the leanest or largest body compositions benefited to a lesser extent from high levels of physical activity.

### 3.4. Physical Activity and Breast Cancer Risk Overall, and Based on Menopausal Status and Body Composition

**Figure 2:**
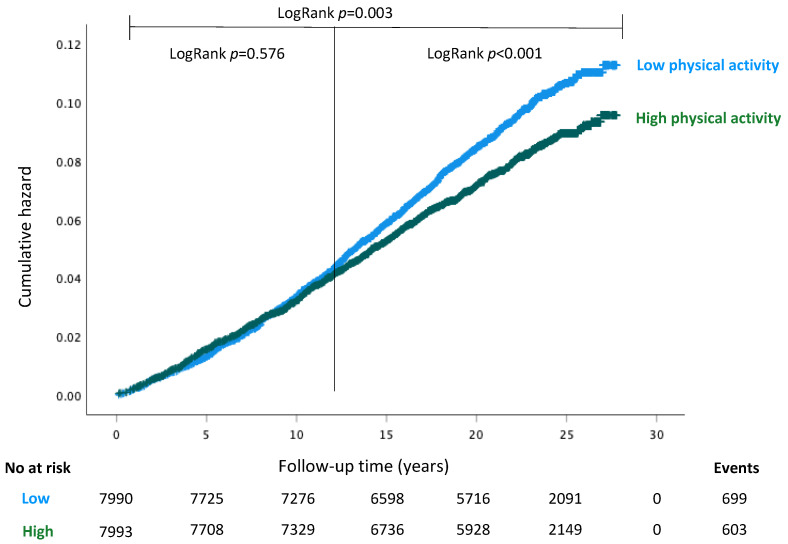
Kaplan–Meier estimates of cumulative hazard for incident breast cancer for all women with LogRank tests presented for the entire follow-up period, and for the short- (0–11 years) and long-term (12+ years) follow-up periods separately.

The cumulative hazard of invasive breast cancer between women with different physical activity levels was estimated with Kaplan–Meier curves (Figure 2). While the high physical activity group had an overall lower breast cancer hazard (LogRank *p* = 0.002), the hazards of low and high physical activity groups followed a non-proportional pattern which had a negligible difference in the early period (0–11 years; LogRank *p* = 0.576), whereas there were evident differences in the late period (12+ years; LogRank *p* < 0.001).

**Figure 3:**
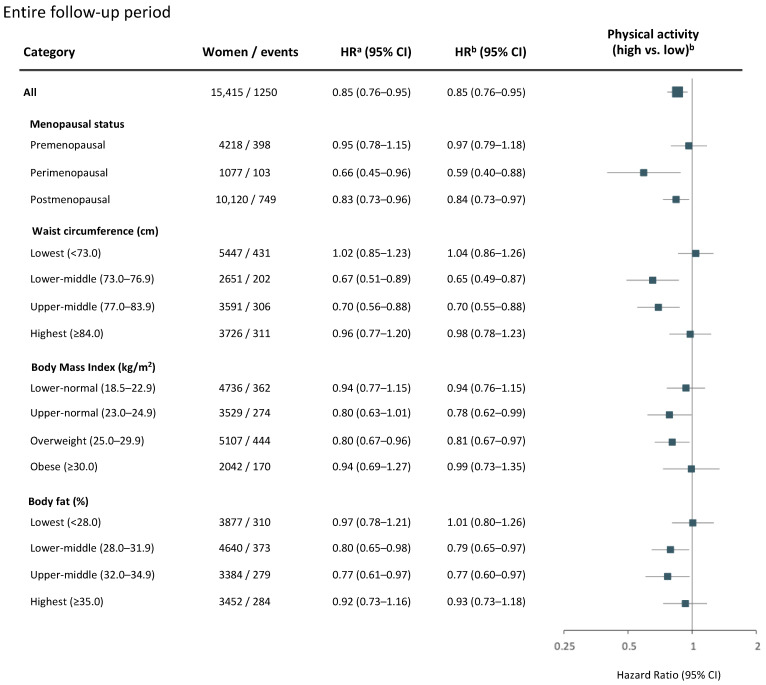
Physical activity and risk of breast cancer over the entire study follow-up. Age- and multivariable-adjusted hazard ratios (HR) with 95% CI for a high versus low level of physical activity among all women, and by menopausal status or body composition. Women and breast cancer events shown represent the numbers available in the multivariable model. The forest plot illustrates multivariable-adjusted HR with 95% CI. ^a^ Model adjusted for age at baseline. ^b^ Multivariable model adjusted for age at baseline, age at menarche, parity, age at first childbirth, oral contraceptive use, current hormone replacement therapy use, socioeconomic index, and alcohol consumption. Models for all women or stratified by body composition (waist circumference, BMI, or body fat percentage) were additionally adjusted for age at menopause.

**Figure 4:**
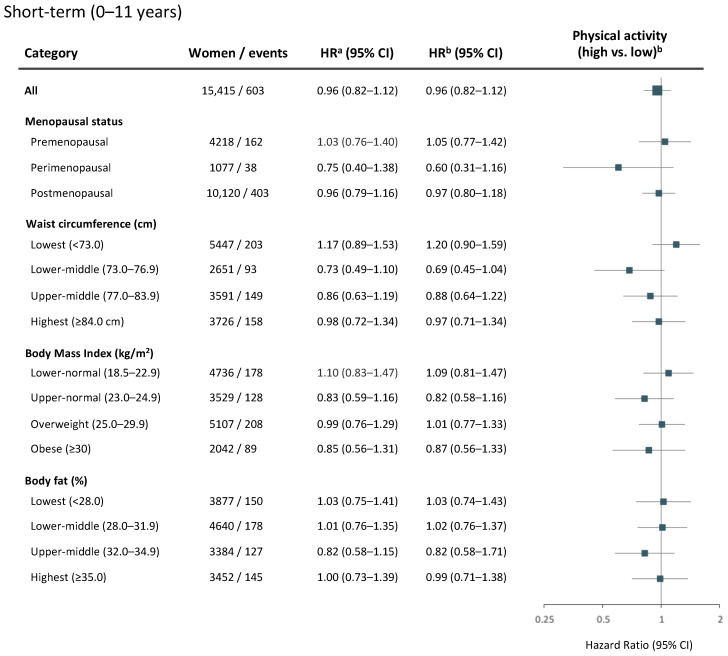
Physical activity and short-term (0–11 years) risk of breast cancer. Age- and multivariable-adjusted hazard ratios (HR) with 95% CI for a high versus low level of physical activity among all women, and by menopausal status or body composition. Women and breast cancer events shown represent the numbers available in the multivariable model. The forest plot illustrates multivariable-adjusted HR with 95% CI. ^a^ Model adjusted for age at baseline. ^b^ Multivariable model adjusted for age at baseline, age at menarche, parity, age at first childbirth, oral contraceptive use, current hormone replacement therapy use, socioeconomic index, and alcohol consumption. Models for all women or stratified by body composition (waist circumference, BMI, or body fat percentage) were additionally adjusted for age at menopause.

**Figure 5:**
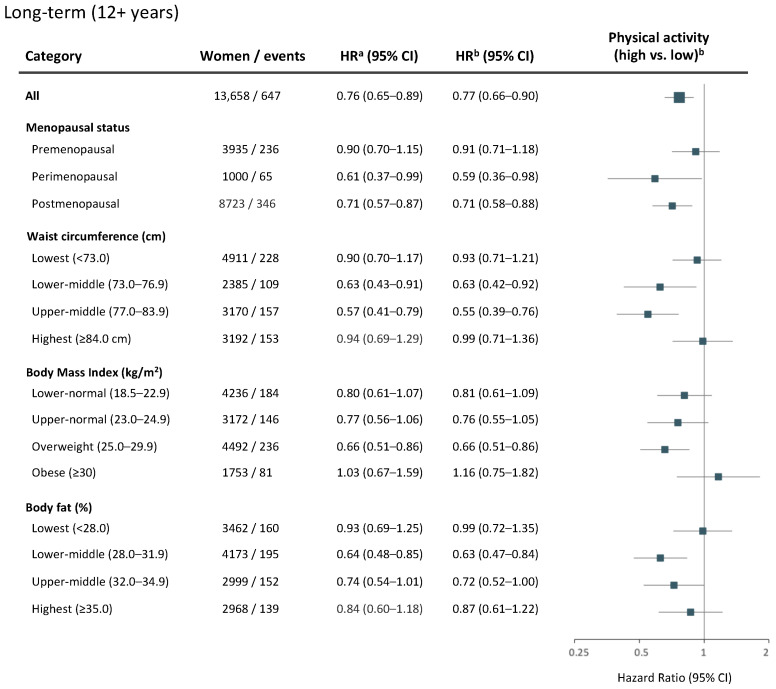
Physical activity and long-term (12+ years) risk of breast cancer. Age- and multivariable-adjusted hazard ratios (HR) with 95% CI for a high versus low level of physical activity among all women, and by menopausal status or body composition. Women and breast cancer events shown represent the numbers available in the multivariable model. The forest plot illustrates multivariable-adjusted HR with 95% CI. ^a^ Model adjusted for age at baseline. ^b^ Multivariable model adjusted for age at baseline, age at menarche, parity, age at first childbirth, oral contraceptive use, current hormone replacement therapy use, socioeconomic index, and alcohol consumption. Models for all women or stratified by body composition (waist circumference, BMI, or body fat percentage) were additionally adjusted for age at menopause.

Age-adjusted and multivariable-adjusted Cox regression analyses provided similar hazard ratios of breast cancer risk by high levels of physical activity (Figure 3, Figure 4 and Figure 5). Across the entire follow-up period, women overall benefited from a 15% lower breast cancer risk (HR_adj_ = 0.85, 95% CI 0.76–0.95) with high compared to low levels of physical activity (Figure 3).

Due to the discrepant effects observed in the early vs. late follow-up periods, separate regression analyses were conducted according to the short-term (0–11 years) and long-term (12+ years) benefits of physical activity. In the short term, no clear associations between physical activity and breast cancer risk were observed overall (HR_adj_ = 0.96, 95% CI 0.82–1.12), or within subgroups by menopausal status or body composition (Figure 4).

In the long term, a 23% lower breast cancer risk (HR_adj_ = 0.77, 95% CI 0.66–0.90) was observed for women overall (Figure 5). With regard to time in life, the most pronounced effects of high physical activity were gained by perimenopausal (HR_adj_ = 0.59, 95% CI 0.36–0.98) and postmenopausal (HR_adj_ = 0.71, 95% CI 0.58–0.88) women. No association between physical activity and breast cancer risk was observed for premenopausal women (Figure 5). With regard to body composition, high physical activity was associated with lower long-term breast cancer risk among women with lower-middle (HR_adj_ =  0.63, 95% CI 0.42–0.92) and upper-middle (HR_adj_ =  0.55, 95% CI 0.39–0.76) waist circumferences, upper-normal (HR_adj_ =  0.76, 95% CI 0.55–1.05) and overweight (HR_adj_ =  0.66, 95% CI 0.51–0.86) BMI levels, and lower-middle (HR_adj_ = 0.63, 95% CI 0.47–0.84) and upper-middle (HR_adj_ = 0.72, 95% CI 0.52–1.00) body fat percentages (Figure 5). No strong associations between physical activity and breast cancer risk were found in either follow-up period among women having the leanest or largest body compositions (Figure 3, Figure 4 and Figure 5).

## 4. Discussion

In this study, the association between physical activity and breast cancer risk in relation to the timing of physical activity in life and body composition were investigated by making use of a prospective cohort with a median follow-up period of 23.2 years. High levels of physical activity that corresponds to >1 h of daily walking per week (≥28.5 MET-h/week) were found to be associated with a 23% lower risk of developing breast cancer. The greatest long-term benefits (12+ years) were observed among women who engaged in high levels of physical activity during or after menopause, or if they had larger body composition with lower-middle to upper-middle waist circumference or body fat percentage, or upper-normal-to-overweight BMI. High physical activity alone did not modify the breast cancer risk for premenopausal women or women with obesity and the largest body composition.

The observed preventive effects of high physical activity on overall breast cancer risk are in agreement with previous studies. A meta-analysis of 31 studies with 63,786 cases found a 12% reduction in breast cancer risk among women in the 75th percentile of physical activity compared to the 25th percentile [26]. A pooled analysis of 10 prospective cohort studies showed a 10% reduction in breast cancer risk from the 10th percentile of physical activity to the 90th percentile [13]. Another pooled analysis made use of MET-hours and showed that moderate-intensity physical activity, equivalent to 15 MET-h/week, could reduce breast cancer risk by 10% [21]. Differences in the age of the study participants and physical activity categories may explain the lesser reduction compared to our study. The cut-off value of 28.5 MET-h/week that was utilized to dichotomize low and high physical activity levels in our study population overlaps with the upper range of physical activity recommendations for adults by the WHO [27] when converted to MET-hours. Notably, a graded reduction in breast cancer risk with higher physical activity (0–30 MET-h/week) has been shown [28], with comparable overall risk reductions as the results of the present study.

With regard to timing in life, the greatest long-term benefits of physical activity in our study were observed among women who engaged in high levels of physical activity during or after menopause. Although perimenopausal women had similar reduced breast cancer risk in the short term (0–11 years), the evidence was more strongly associated in the long term (12+ years). The association between high physical activity and lower breast cancer risk for postmenopausal women was only seen in the long term. A study of the NIH-AARP Diet and Health Study cohort showed a 13% risk reduction in the most active postmenopausal women compared to inactive women [29]. Another study of the same cohort showed a 16% risk reduction among the postmenopausal women who engaged in 7 h/week of moderate-to-vigorous activity compared to inactive women [30]. While these studies provide a large number of participants (182,862), the physical activity assessments were not based on MET-hours, and instead relied on different assessment methods which did not take energy expenditures into consideration. In a separate study, no association was found between physical activity and breast cancer risk among premenopausal women, consistent with the present findings [31]. Recent results from the UK Biobank found high physical activity to be associated with lower breast cancer risk among both pre- and postmenopausal women [16]. While the protective association in premenopausal women was only evident for women in the top quartile (≥58.3 MET-h/week), postmenopausal women with physical activity levels above the median (≥29.6 MET-h/week) had a reduced breast cancer risk, in line with our findings.

We also examined the effects of physical activity on breast cancer risk in relation to anthropometric measures to provide further insights into the physical activity and breast cancer association with regard to body composition and adiposity. Our study showed that women with lower-middle to upper-middle waist circumference (corresponding to European clothing sizes M–L) or body fat percentage (fair–poor), or upper-normal-to-overweight BMI (23.0–24.9 and 25.0–29.9 kg/m^2^) benefited the most from physical activity regarding reduced long-term breast cancer risk. On the contrary, the women having the lowest and highest waist circumference, body fat percentage, or BMI did not have a modified breast cancer risk upon high physical activity alone. Our findings are similar to the results of a meta-analysis of 18 cohort studies that showed a 15% and 14% of reduction in breast cancer risk in women who had a BMI of <25 kg/m^2^ and ≥25 kg/m^2^, respectively, and engaged in high compared to low levels of physical activity [32].While no risk reduction was observed in premenopausal women, the risk reduction was found to be 13% for postmenopausal women for both BMI groups. Similar to our findings, women who had a BMI of ≥30 kg/m^2^ did not benefit from a risk-reducing effect of physical activity [20,32]. When 22 studies on BMI and risk reduction were examined, it was found that women with low normal BMI (<22 kg/m^2^) benefited with a risk reduction of 27% from physical activity, whereas women with upper-normal (22–25 kg/m^2^), high (≥25 kg/m^2^), and very high (≥30 kg/m^2^) BMI benefited with risk reductions of 24%, 18%, and <1%, respectively [19]. While the data regarding upper-normal and higher BMIs are consistent with our findings, the high level of risk reduction in women with the lowest BMI could partly be attributed to the different definition of low BMI or physical activity cut-offs used in these studies compared to ours. Moreover, our results in relation to BMI are corroborated by similar findings with regard to waist circumference and body fat percentages in the present study.

The biological mechanisms and the causal links between physical activity and breast cancer risk reduction are yet to be established. For pre- and perimenopausal breast cancer risk, physical activity could be exerting its risk-reducing effect via its modulation of estrogen. For postmenopausal breast cancer risk, a decrease in adiposity, thus a decrease in estrogen and insulin signaling, could be responsible for physical activity-mediated risk reduction. Recently, exercise was shown to be negatively correlated with circulating metabo-inflammatory biomarkers C-reactive protein, IL-6, and leptin, while positively correlated with adiponectin and the estrogen-regulator sex hormone binding globulin (SHBG) [33]. To gain a better understanding of the biological mechanisms behind this, the associations between low- and high-intensity physical activities and breast cancer risk can be addressed in a subsequent study with regard to menopausal status.

The strengths of our study include the large study population with prospective design and long follow-up. Another strength is the availability of physical activity data comprising time spent for 17 different activities, and the combination of duration and intensity. A further strength is the comprehensive data regarding breast cancer risk factors which enabled us to perform multivariable adjustments in regression analyses. On the other hand, the limitations in our study include that the physical activity data were reported retrospectively in the baseline questionnaire for the year prior to study inclusion and not reported during the follow-up period. Therefore, it is unknown whether the participants continued the same level of physical activities that they initially reported at baseline. Although adjusting for known and potential breast cancer risk factors, it cannot be fully excluded that the remaining unknown or residual confounders could influence the results. Another limitation is the lack of information regarding body compositions at the end of the follow-up period, which could potentially have improved the interpretation of the results.

## 5. Conclusions

Physical activity is beneficial for overall health and chronic disease prevention. Although the overall breast cancer risk-reducing effect of physical activity is previously known, it is important to identify the optimal time in life and which groups this effect is exerted upon the most. In this study, we showed that women during or after menopause, or with upper-normal-to-overweight body compositions, benefit from physical activity the most. For premenopausal women with the leanest or largest (obese) body compositions, no evident modified risk was found which implies that these women may potentially benefit from additional risk-reduction measures. Further research needs to be conducted to elucidate the associations between breast cancer prevention and lifestyle changes for women with large body compositions at an increased risk of breast cancer. The associations between physical activity-mediated breast cancer risk reduction and breast tumor characteristics should also be examined to gain an in-depth understanding of the benefits of physical activity.

## Figures and Tables

**Table 1:** Distribution of established and potential risk factors for breast cancer at baseline among included and excluded women.

		Physical Activity Level		
	All	Low	High	Not Included
	(*n* = 17,035)	(*n* = 7990)	(*n* = 7993)	(*n* = 1052)
Age at baseline	56.7 (50–63.8)	56.6 (50.0–63.3)	56.3 (49.8–63.8)	61 (53.8–66)
Age at diagnosis	64.6 (56.2–72)	68.5 (62.4–74.2)	67.2 (61.6–74.0)	52.7 (46.7–60.4)
**ANTHROPOMETRY**				
Weight (kg)	66 (60–74)	67 (60–75)	66 (60–73)	68 (61–77)
Height (cm)	164 (160–168)	163 (159–168)	164 (160–168)	163 (159–167)
Waist (cm)	76 (70–83)	77 (71–85)	75 (70–82)	78 (72–87)
Hip (cm)	97 (91–103)	97 (92–104)	96 (91–102)	98 (93–106)
BMI (kg/m^2^)	24.7 (22.5–27.7)	24.9 (22.7–28.0)	24.4 (22.3–27.1)	25.5 (22.9–28.7)
Body fat (%)	31 (28–34)	31 (28–35)	30 (27–34)	32 (29–35)
**SOCIOECONOMY**				
Occupation				
Manual worker	6430 (38.1)	3098 (39.2)	2879 (36.3)	453 (44.2)
Nonmanual worker	9156 (54.3)	4226 (53.4)	4434 (55.9)	496 (48.4)
Employer/self-employed	1276 (7.6)	587 (7.4)	614 (7.7)	75 (7.3)
Missing	173 (1.0)	79 (1.0)	66 (0.8)	28 (2.7)
**LIFESTYLE AND REPRODUCTIVE FACTORS**				
Age at menarche				
≤12 years	3726 (22)	1778 (22.4)	1739 (21.9)	209 (20.3)
13–14 years	9013 (53.3)	4203 (52.9)	4275 (53.9)	535 (51.8)
>15 years	4171 (24.7)	1963 (24.7)	1920 (24.2)	288 (27.9)
Missing	125 (0.7)	46 (0.6)	59 (0.7)	20 (1.9)
Parity				
0	2184 (13.0)	968 (12.3)	1046 (13.3)	170 (16.5)
1	3640 (21.7)	1729 (22.0)	1635 (20.8)	276 (26.8)
2	6990 (41.7)	3283 (41.8)	3350 (42.6)	357 (34.7)
3	2813 (16.8)	1339 (17.0)	1329 (16.9)	145 (14.1)
4 or more	1118 (6.7)	540 (6.9)	498 (6.3)	80 (7.8)
Missing	290 (1.7)	131 (1.6)	135 (1.7)	24 (2.3)
Age at first childbirth				
Nulliparous	2184 (13.1)	968 (12.3)	1046 (13.3)	170 (16.6)
≤20 years	2832 (16.9)	1379 (17.6)	1278 (16.3)	175 (17.1)
21–25 years	5970 (35.7)	2772 (35.3)	2856 (36.4)	342 (33.4)
26–30 years	4151 (24.8)	1952 (24.9)	1962 (25.0)	237 (23.1)
>30 years	1596 (9.5)	783 (10.0)	712 (9.5)	101 (9.9)
Missing	302 (1.8)	136 (1.7)	139 (1.7)	27 (2.6)
Oral contraceptive use				
Never	8665 (50.9)	4016 (50.3)	4022 (50.4)	627 (60.1)
Ever	8354 (49.1)	3972 (49.7)	3966 (49.6)	416 (39.9)
Menopausal status				
Premenopausal	4452 (26.1)	2110 (26.4)	2189 (27.4)	153 (14.5)
Perimenopausal	1195 (7.0)	553 (6.9)	581 (7.3)	61 (5.8)
Postmenopausal	11,388 (66.9)	5327 (66.7)	5223 (65.3)	838 (79.7)
Current HRT use				
No	13,111 (77.2)	6138 (77.0)	6064 (76.1)	909 (86.6)
Yes	3877 (22.8)	1829 (23.0)	1907 (23.9)	141 (13.4)
Alcohol				
Nothing last year	1947 (11.5)	966 (12.1)	768 (9.6)	213 (20.6)
Something last year	2054 (12.1)	966 (12.1)	917 (11.5)	171 (16.5)
Something last month	12,997 (76.5)	6046 (75.8)	6299 (78.9)	652 (62.9)
Missing	37 (0.2)	12 (0.2)	9 (0.1)	16 (1.5)
Physical activity				
Activity time (min/week)	376 (225–576)	228 (150–301)	578 (463–760)	368 (214–543)
MET-hours/week	28.3 (17.3–43.5)	17.3 (11.4–22.8)	43.7 (35.0–57.4)	27.5 (16.1–41.3)

Data presented as median (interquartile range; IQR) or total numbers (valid column %). Missing data are shown as total % and not presented if missing <1% in all columns.

**Table 2:** Physical activity levels according to menopausal status or body composition, among all women and by breast cancer status.

	All Women		No Breast Cancer		Breast Cancer	
	min/Week	MET-h/Week	min/Week	MET-h/Week	min/Week	MET-h/Week
**All**	377 (228–578)	28.5 (17.3–43.7)	380 (228–580)	28.6 (17.3–43–8)	353 (222–555)	26.7 (17.0–41.9)
**Menopausal status**						
Premenopausal	371 (228–558)	28.9 (17.8–43.4)	372 (228–555)	29.0 (17.7–43.2)	368 (230–582)	28.2 (18.7–44.6)
Perimenopausal	367 (227–562)	29.0 (17.8–42.8)	373 (230–564)	29.4 (17.9–43.4)	328 (210–501)	24.6 (15.4–39.1)
Postmenopausal	383 (226–590)	28.1 (17.0–43.9)	385 (228–593)	28.3 (17.0–44.1)	348 (220–548)	26.1 (16.3–41.0)
**Waist circumference (cm)**						
Lowest (≤73.0)	408 (253–605)	31.0 (19.4–46.0)	408 (253–605)	31.0 (19.5–46.0)	416 (248–601)	31.0 (19.3–46.5)
Lower-middle (73.0–76.9)	380 (233–576)	29.0 (18.0–43.5)	385 (235–585)	29.4 (18.2–44.0)	330 (213–509)	24.9 (16.8–39.1)
Upper-middle (77.0–83.9)	370 (222–569)	28.0 (17.0–43.0)	376 (223–575)	28.0 (17.1–43.3)	328 (206–508)	24.4 (15.4–38.4)
Highest (≥84.0)	343 (195–536)	25.4 (14.6–40.2)	343 (195–538)	25.5 (14.4–40.2)	338 (212–530)	25.0 (15.7–40.3)
**Body Mass Index (kg/m^2^)**						
Lower-normal (<23.0)	393 (240–595)	30.0 (18.7–45.0)	393 (240–595)	30.1 (18.7–45.2)	378 (229–585)	29.1 (18.3–44.1)
Upper-normal (23.0–24.9)	390 (240–575)	29.3 (18.4–43.8)	393 (240–576)	29.6 (18.4–43.9)	354 (240–553)	26.9 (18.0–41.9)
Overweight (25.0–29.9)	375 (225–588)	28.0 (17.0–44.0)	379 (225–595)	28.1 (17.0–44.5)	343 (215–525)	26.0 (16.3–40.8)
Obese (≥30.0)	330 (183–522)	24.2 (14.0–39.0)	330 (181–520)	24.3 (13.8–38.9)	333 (203–535)	23.7 (15.0–40.3)
**Body fat (%)**						
Lowest (≤28.0)	414 (255–607)	31.7 (20.0–46.6)	414 (255–609)	31.8 (20.0–46.6)	420 (240–603)	31.0 (19.5–46.0)
Lower-middle (28.0–31.9)	388 (240–585)	29.3 (18.4–44.3)	390 (240–588)	29.5 (18.5–44.5)	348 (231–563)	27.1 (18.2–42.2)
Upper-middle (32.0–34.9)	368 (225–564)	27.5 (16.6–43.0)	371 (225–570)	27.8 (16.7–43.6)	338 (213–500)	25.5 (16.2–37.7)
Highest (≥35.0)	340 (188–535)	24.8 (14.0–40.0)	340 (188–535)	25.0 (14.0–40.0)	332 (185–530)	24.2 (14.8–40.6)

Data presented as median (interquartile range; IQR).

**Table 3:** Breast cancer incidence per 100,000 person-years (py) for all women, and according to physical activity level.

	All Women		Low Physical Activity		High Physical Activity		Relative Risk
	Women/Events (n)	BCs/100,000 py	Women/Events (n)	BCs/100,000 py	Women/Events (n)	BCs/100,000 py	(%)
**All**	15,983/1302	387	7990/699	418	7993/603	356	–14.9
**Menopausal status**							
Premenopausal	4299/405	423	2110/205	435	2189/200	412	–5.3
Perimenopausal	1134/109	424	553/64	516	581/45	339	–34.4
Postmenopausal	10,550/788	366	5327/430	399	5223/358	332	–16.7
**Waist circumference (cm)**							
Lowest (≤73.0)	5626/453	373	2496/199	370	3130/254	376	1.6
Lower-middle (73.0–76.9)	2749/210	356	1353/124	429	1396/86	287	–33.1
Upper-middle (77.0–83.9)	3728/316	403	1930/190	472	1798/126	331	–30.0
Highest (≥84.0)	3880/323	414	2211/186	420	1669/137	405	–3.6
**Body Mass Index (kg/m^2^)**							
Lower-normal (<23.0)	4935/383	362	2295/184	375	2640/199	351	–6.3
Upper-normal (23.0–24.9)	3660/288	369	1755/154	413	1905/134	328	–20.6
Overweight (25.0–29.9)	5272/456	415	2696/257	460	2576/199	369	–19.7
Obese (≥30.0)	2116/175	406	1244/104	416	872/71	393	–5.6
**Body fat (%)**							
Lowest (≤28.0)	4024/329	383	1722/144	391	2302/185	377	–3.7
Lower-middle (28.0–31.9)	4791/381	371	2311/204	415	2480/177	331	–20.2
Upper-middle (32.0–34.9)	3510/294	399	1843/173	449	1667/121	345	–23.1
Highest (≥35.0)	3595/293	400	2082/174	414	1513/119	382	–7.8

## Data Availability

Data are available to appropriate academic parties upon reasonable request to the Malmö Diet and Cancer Study steering committee.
